# Circulating *hsa-miR-221* as a possible diagnostic and prognostic biomarker of diabetic nephropathy

**DOI:** 10.1007/s11033-023-08846-y

**Published:** 2023-10-13

**Authors:** Marwa Sayed Abdel-Tawab, Mohamed Gamal Mohamed, Noha A. Doudar, Enas Ezzat Rateb, Hoda Ramadan Reyad, Naglaa Adli Abd Elazeem

**Affiliations:** 1 Medical Biochemistry Department, Faculty of Medicine, Beni-Suef University, Beni-Suef, Egypt; 2 Internal Medicine Department, Faculty of Medicine, Beni-Suef University, Beni-Suef, Egypt; 3 Clinical and Chemical Pathology Department, Faculty of Medicine, Beni-Suef University, Beni-Suef, Egypt; 4 Physiology Department, Faculty of Medicine, Beni-Suef University, Beni-Suef, Egypt

**Keywords:** Diabetes mellitus, Diabetic nephropathy, *hsa-miR-221*

## Abstract

**Background:**

Diabetic nephropathy (DN), which is a chronic outcome of diabetes mellitus (DM), usually progresses to end-stage renal disease (ESRD). The DN pathophysiology, nevertheless, is not well-defined. Several miRNAs were reported to be either risk or protective factors in DN.

**Methods, and results:**

The present study sought to inspect the potential diagnostic and prognostic value of *hsa-miR-221* in DN. The study included 200 participants divided into four groups: Group 1 (50 patients with DN), Group 2 (50 diabetic patients without nephropathy), Group 3 (50 nondiabetic patients with CKD), and Group 4 (50 healthy subjects as a control group). Patients in groups 1 and 3 were further classified based on the presence of macroalbuminuria and microalbuminuria. *Hsa-miR-221* expression was measured by RT- qRT-PCR. DN patients had significantly elevated serum *hsa-miR-221* levels than the other groups, while diabetic patients without nephropathy exhibited elevated levels compared to both nondiabetic patients with CKD, and the control group. The DN patients with macroalbuminuria revealed significantly higher mean values of *hsa-miR-221* relative to the patients with microalbuminuria. Significant positive associations were observed in the DN group between serum *hsa-miR-221* and fasting insulin, fasting glucose, HOMA IR, ACR, and BMI. The ROC curve analysis of serum *hsa-miR-221* in the initial diagnosis of DN in DM revealed high specificity and sensitivity.

**Conclusions:**

It is concluded that *hsa-miR-221* has the potential to be a useful biomarker for prognostic and diagnostic purposes in DN.

## Introduction

 Between 1980 and 2021, the number of diabetic patients globally increased from 108 million to 536.6 million, climbing to 783.2 million in 2045. Type 2 diabetes represents about 90–95% of these cases [1, 2].

Micro- and macrovascular complications in diabetes mellitus (DM) affect various systems and organs [3]. Diabetic nephropathy (DN) is among the most serious and common diabetes complications and develops in 20% of diabetic patients [4, 5]. Renal replacement would be necessary for approximately half of all chronic diabetes patients having end-stage renal disease (ESRD) [6].

Diabetes is considered responsible for 80% of ESRD cases worldwide. Type 2 diabetes is responsible for 91% of all new cases of diabetes related ESRD, following the United States Renal Data System (USRDS) [7].

Proteinuria and glomerulopathy are developed gradually with years-long latent periods of DN [5]. Although substantial recent studies, the specific etiology of DN remains unknown. However, the development of DN is known to involve various variables and mechanisms.

The pathophysiology of DN is marked by hypertrophy and hyperplasia of the mesangium of the glomeruli and dysfunction of podocyte [8]. Long-term oxidative stress, advanced glycation end products (AGEs), hyperglycemia, and inflammation are all contributors to the development of DN [9].

MicroRNAs (miRs) have been attributed to the DN pathogenesis in two ways: Binding upregulated miRs to the protective genes in the kidney, repressing them, and downregulated miRNAs, in turn, can cause upregulation of genes that promote the mesangial expansion of or podocyte dysfunction [10].

*Hsa-MiRNA-221* (*Hsa-miR-221*) accounts for a miRNA recognized within human cells of endothelium that regulate angiogenesis [11]. *Hsa-miR-221* is an X-chromosome gene associated with metabolic disease [12].

Previous studies on *hsa-miR-221* reported that its expression was upregulated and involved in the physiopathology of diabetes and type 2 diabetes (T2D) macrovascular and microvascular complications via targeting specific genes [13].

It has been found that aberrant overexpression of *hsa-miR-221* accelerates retinal neovascularization in diabetic retinopathy by promoting cell migration, apoptosis, and proliferation, boosting hyperplasia and retinal artery blockage, and causing retinal ischemia and hypoxia [14].

As we know so far, no research has been accomplished to examine the potential of serum *hsa-miR-221* as a biomarker for prognostic and diagnostic purposes in DN. The current study explores the hypothesis based on earlier research: whether serum *hsa-miR-221* constitutes an innovative biomarker with significant accuracy for the diagnosis of DN.

## Subjects and methods

### Study design

This case-control study was conducted on a total of 200 participants chosen from the Internal Medicine Department at Beni-Suef University Hospital. The participants were divided into four groups: Group 1 (50 diabetic nephropathy patients), Group 2 (50 diabetic patients without nephropathy), Group 3 (50 nondiabetic patients having chronic kidney disease (CKD), and Group 4 (50 apparently healthy subjects as a control group). Then, the fifty patients with diabetic nephropathy were subdivided into 27 patients with macroalbuminuria and 23 patients with microalbuminuria. In contrast, the 50 patients with nondiabetic CKD were subdivided into 20 patients with macroalbuminuria and 20 patients with microalbuminuria.

### Ethical considerations

The current study’s protocol followed the ethical principles of the Helsinki Declaration [15]. The study was granted approval by the Ethical Committee of the Faculty of Medicine, Beni-Suef University, with approval number (FMBSUREC /09072023/Abd Elazeem). Informed written consent was obtained from all participants.

### Patients’ selection

According to the American Diabetes Association, both diabetic patients [16] and patients with diabetic nephropathy [17] were diagnosed. DN criteria of diagnosis were chronic albuminuria (> 200 g/min or > 300 mg/d) demonstrated on at least two occasions at least 3–6 months apart or an albumin-to-creatinine ratio (ACR) > 300 mg/g, with a consistent reduction in the glomerular filtration rate (GFR), without symptoms or signs of other primary renal diseases.

All patients’ clinical and medical histories were obtained. Next, the patients were submitted to a comprehensive clinical evaluation to select individuals with diabetic nephropathy who were over the age of 18. Exclusion criteria were hypertension, congestive heart failure, severe illness, current infection, peripheral artery disease, pregnancy, and malignancy. By measuring weight and height, body mass index (BMI) was estimated [18].

### Sampling and laboratory investigations

Five milliliters of venous blood were drawn and put in either plain or fluoride-containing vacutainer tubes. Then, Urine samples were collected to evaluate albuminuria and estimate the urinary ACR and eGFR. Following overnight fasting for 10 h at least, biochemical tests (fasting insulin, fasting glucose, total cholesterol, HDL-c, LDL-c, HbA1c, triglycerides, creatinine, and urea) were conducted. The formula [19] adopted to assess the Homeostasis model assessment for insulin resistance (HOMA-IR) was as follows: HOMA-IR = fasting insulin (mlU/L) X fasting glucose (mg/dL) ÷ 405. Insulin resistance was deemed high if it was ˃ 2 [20]. Additionally, the Chronic Kidney Disease Epidemiology Collaboration (CKD-EPI) equation [21] was employed to determine eGFR. LDL-c levels were calculated by Friedewald equation [22]: LDL-c = (Total Cholesterol mg/dL) − (HDL-c mg/dL) − (TGs mg/dL /5).

### Kits used for investigations

All laboratory tests were performed according to the instructions of the manufacturer’s protocols for the following kits: Human Insulin ELISA Kit (Cat NO.:SL0933Hu, SUNLONG, China), Human Hb A1c ELISA Kit (Cat No: SL3471Hu, SUNLONG, China), Glucose Colorimetric Assay (Cat No: KitMAK263-1KT, Sigma-Aldrich, Germany), Triglyceride Colorimetric Assay Kit (Cat No: 10,010,303, Cayman, USA), Cholesterol Quantitation Kit Cat No: MAK043-1KT, Sigma-Aldrich, Germany), HDL-c Colorimetric Assay Kit (Cat No: E-BC-K222-S, Elabscience, USA), BUN Colorimetric Assay Kit (Cat No: MAK006-1KT, Sigma-Aldrich, Germany), Creatinine Colorimetric Assay Kit (Cat No: MAK080, Sigma-Aldrich, Germany), and ACR colorimetric Assay Kit (Cat NO: LS-K562-100, LSBio, UK).

### Extraction of miRNA

Purified total RNA, comprising miRNA, from serum samples was extracted using the miRNeasy mini kit (cat. no. 217,004, QIAGEN, Germany) following the manufacturer’s instructions. Spectrophotometry (JENWAY, USA) was utilized to measure the isolated RNA at 260 nm.

### Real time quantitative reverse-transcription polymerase chain reaction (qRT-PCR)

According to manufacturer’s instructions, Real-time qRT-PCR was accomplished utilizing a single-plex TaqMan two-step stem-loop real-time RT. TaqmanTM miRNA assay and the TaqmanTM miRNA Reverse Transcription Kit (Thermo Fisher Scientific, Waltham, MA, USA) were utilized to produce cDNA. The RT reaction total volume was 15 µL. Primer Premier 6.00 was used to design the primers (Premier Biosoft, Palo Alto, CA, USA). The micro-RNA specific stem loop primer of miRNA-221, and miRNA-16 (a reference gene) were added. TaqMan^®^ Small RNA Assay, and the TaqMan^®^ Universal PCR Master Mix II PCR were used to obtain amplified PCR from cDNA.

### Analysis of data

The cycle threshold (CT) was adopted to quantify the levels of miRNA-221 expression. The comparative CT method (ΔΔ CT) was calculated by the difference between miRNA-221 CT and the reference gene average CT in each sample. Furthermore, miRNA expression fold change was calculated according to the formula 2^−ΔΔCt^ [23] using the controls as calibrators.

### Statistical analysis

SPSS (Statistical Package for Social Sciences) version 23 (SPSS Inc., Chicago, IL, USA) was used. The data were presented as mean ± SD. The statistical analysis of group differences involved the use of the Kruskal–Walli test, the Shapiro–Wilk test, and the t test. To compare the discrepancies among groups, Tukey post-hoc testing was employed. To evaluate the linear relationships between the study genes and clinical factors, a simple linear correlation (Pearson correlation coefficient test) (r) was conducted. If p was 0.05, the p value was considered significant. Receiver operating characteristic curves (ROC curves) were employed to appraise the diagnostic performance of the studied parameter.

## Results

The Frequency of some demographic, and clinical data in the studied groups were demonstrated in Table 1.

**Table 1:** The Frequency of some demographic, and clinical data in the studied groups

Parameters	DN N = 50 N/%	DM N = 50 N/%	ND CKD N = 50 N/%	Controls N = 50 N/%
Gender				
Male	28/56%	29/58%	31/62%	33/66%
Female	22/44%	21/42%	19/38%	17/34%
Obesity				
Non-Obese	10/20%	27/54%	26/52%	42/84%
Overweight	33/66%	17/34%	20/40%	6/12%
Obese	7/14%	6/12%	4/8%	2/4%
Albuminuria				
Micro-albuminuria	23/46%	−/−	30/60%	−/−
Macro-albuminuria	27/54%	−/−	20/40%	−/−
Normal	−/−	50/100%	−/−	50/100%
Treatment with antidiabetic drugs				
Rosiglitazone	24/48%	22/44%	−/−	−/−
Glyburide	26/52%	14/28%	−/−	−/−
Metformin	−/−	14/28%	−/−	−/−

*DN* diabetic nephropathy, *DM* diabetes mellitus without nephropathy, *ND CKD* Nondiabetic chronic kidney disease, *N* number of cases, *%* percentage of frequency

### The mean levels of demographic and clinical data in the studied groups

The mean levels of demographic and clinical data in the investigated groups were recorded in Table 2.

**Table 2:** The demographic, and clinical data of the subjects in the studied groups

Parameters	DN (N = 50) M ± SD	DM (N = 50) M ± SD	ND CKD (N = 50) M ± SD	Controls (N = 50) M ± SD
Age (years)	48.1 ± 5.5	50.2 ± 5.1	50.6 ± 7.1	50.2 ± 6.4
BMI (kg/m^2^)	26.6 ± 2.6 (b c d) #	25 ± 2.6 (ad) #	24.8 ± 2.2 (ad) #	22.7 ± 2.8 (a b c) #
Duration of disease (years)	15.3 ± 1.5 (b c) #	7.7 ± 1.7 (a c) #	6.3 ± 2.3 (a b) #	
HBA1c %	8.5 ± 0.6 (b c d) #	7.4 ± 0.5 (a c d) #	4.7 ± 0.5 (a b) #	4.5 ± 0.4 (a b) #
Fasting Insulin** (**mU/L)	22.1 ± 3.1 (b c d) #	13.8 ± 2.8 (a c d) #	5.4 ± 1.7 (a b) #	5 ± 1.2 (a b) #
Fasting glucose (mg/dL)	172.8 ± 13.8 (b c d) #	202 ± 24.6 (a c d) #	80.4 ± 7.5 (ab) #	79.2 ± 6.1 (a b) #
HOMA-IR	9.4 ± 1.6 (b c d) #	6.9 ± 1.5 (a c d) #	1.1 ± 0.4 (a b) #	0.98 ± 0.2 (a b) #
Serum TG (mg/dL)	214.9 ± 21.2 (c d) #	208 ± 23.9 (c d) #	125 ± 21.3 (a b) # d*	112.5 ± 11.7 (a b) # c*
Serum cholesterol (mg/dL)	175.9 ± 6.5 (c d) #	174.1 ± 9.2 (c d) #	166.8 ± 11.6 (a b) #	166 ± 12.1 (a b) #
Serum LDL-C (mg/dL)	108.2 ± 15.3	104 ± 6.6	107.6 ± 8.3	107.3 ± 8
Serum HDL-C (mg/dL)	43.5 ± 2.3 (c d) #	45.2 ± 3.3 (c d) #	54.2 ± 9.5 a# b*	63.7 ± 1.9 (a b) #
Urinary ACR (mg/g)	507.56 ± 287 (b c d) #	24.4 ± 3.2 (a c) #	243.5 ± 178 a # (b d) *	16.6 ± 3.3 (a c) #
eGFR (mL/min/1.73 m^2^)	35.5 ± 11.7 (b c d) #	76 ± 3.44 (a c d) #	60.3 ± 28.8 (a b d) #	96.5 ± 1.8 (a b c) #
BUN (mg/dL)	54.4 ± 8.6 (b d) #	20.9 ± 2.8 (a c) #	51.5 ± 8 (b d) #	18.5 ± 2.9 (a c) #
Serum creatinine (mg/dL)	2.1 ± 0.3 (b c d) #	0.8 ± 0.04 (a c) # d*	1.24 ± 0.12 (a b d) #	0.73 ± 0.02 (ac) # b*

*DN* diabetic nephropathy, *DM* diabetes mellitus without nephropathy, *ND CKD* Nondiabetic chronic kidney disease, *N* number of subjects, *M ± SD* mean **±** standard deviation, *a* Significant differences compared to diabetic nephropathy group, *b* Significant differences compared to DM without nephropathy, *c* Significant differences compared to Nondiabetic CKD, *d* Significant differences compared to controls

*Significant difference (p value < 0.05), #High significant difference (p value < 0 0.001)

The current study was conducted on 121 males (60.5% of subjects) and 79 females (39.5% of subjects). The mean levels of age and LDL-c revealed no significant variations across the studied groups.

In diabetic nephropathy, the mean levels of BMI, serum fasting insulin, serum fasting glucose, HOMA IR, serum HbA1c, serum creatinine, and ACR exhibited significantly higher mean levels. In contrast, the eGFR exhibited significantly lower mean levels than diabetic patients without nephropathy, nondiabetic patients with CKD, and healthy controls. Diabetic nephropathy patients had a significantly longer disease duration than diabetic patients without nephropathy and nondiabetic patients with CKD.

Compared to healthy controls and nondiabetic patients with CKD, both diabetic nephropathy and diabetic patients without nephropathy showed a significant decline in mean serum HDL-c levels along with significant increases in mean serum levels of TG and cholesterol.

The mean levels of serum BUN demonstrated a significant increase in diabetic nephropathy compared to diabetic patients without nephropathy, healthy controls, and nondiabetic patients having CKD.

### The mean level of serum *hsa-miR-221 *in the studied groups

**Fig. 1:**
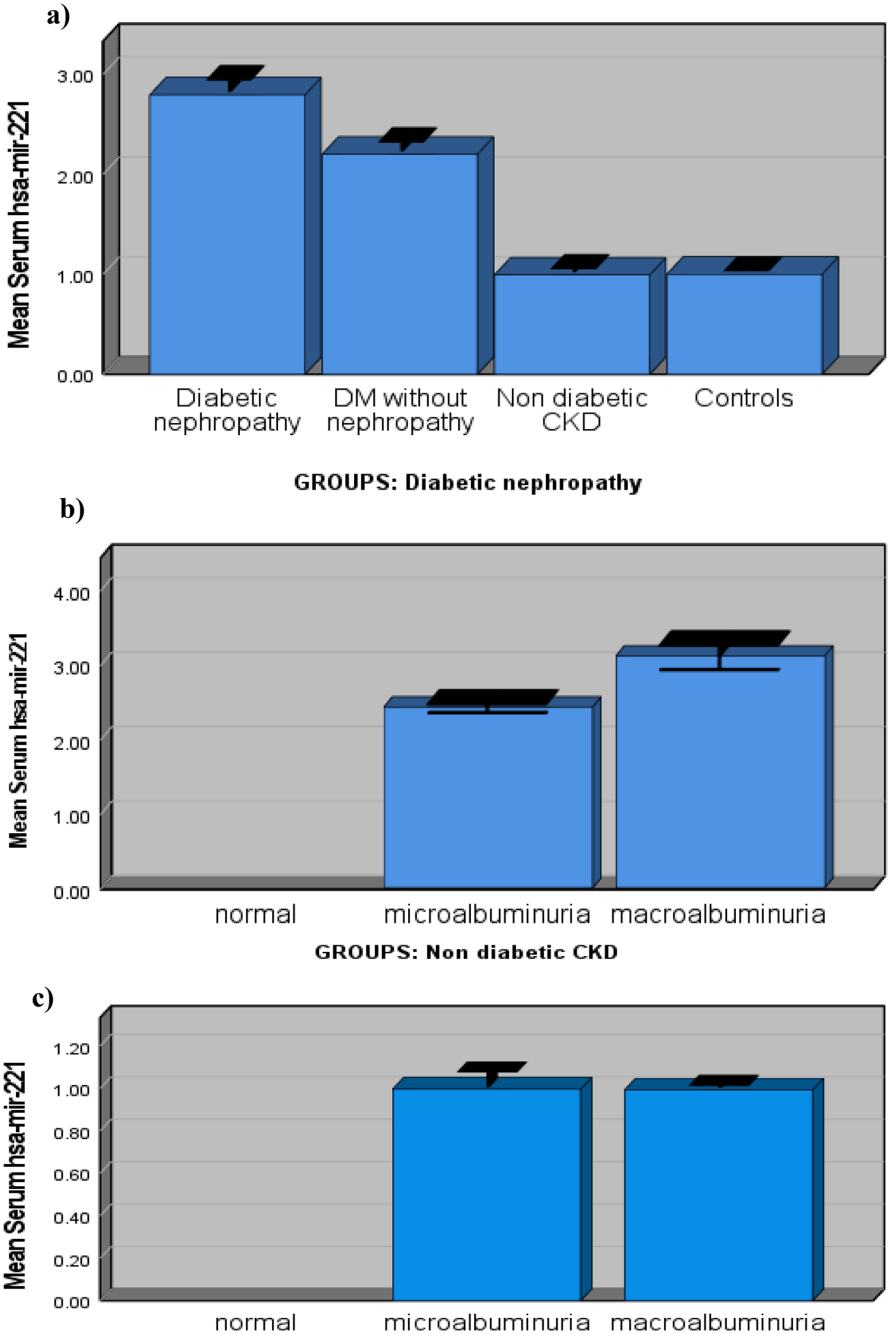
The mean levels of serum *hsa-mir-221* in studied subjects. **a** mean levels of serum *hsa-mir-221* in studied groups. **b** mean levels of serum *hsa-mir-221* in subjects with different degrees of albuminuria in diabetic nephropathy group. **c** mean levels of serum *hsa-mir-221* in subjects with different degrees of albuminuria in nondiabetic CKD group

As demonstrated in Fig. 1a, the mean levels of serum *hsa-miR-221* were significantly (p value ˂0.001) higher in diabetic nephropathy patients (3.06 ± 0.44) than those in diabetic patients without nephropathy (2.19 ± 0.39), nondiabetic patients with CKD (0.995 ± 0.16), and healthy controls (1 ± 0.043). They were significantly (p-value ˂0.001) higher in diabetic patients without nephropathy (2.19 ± 0.39) than those in nondiabetic patients with CKD (0.995 ± 0.16) and healthy controls (1 ± 0.043); however, they were comparable in nondiabetic patients with CKD (0.995 ± 0.16) and healthy subjects (1 ± 0.043).

### The mean levels of *hsa-miR-221 *in the subgroups

According to Table 3, the mean levels of *hsa-miR-221* in males and females in each group were not significantly different. Additionally, they showed no significant variations between patients who used rosiglitazone, glyburide, or metformin in any group of diabetic patients, However, they were significantly higher as obesity developed in patients with diabetic nephropathy.

**Table 3:** Serum *hsa-mir-221* levels in the subgroups of the studied groups

Subgroups	DN M ± SD	DM M ± SD	ND CKD M ± SD	Controls M ± SD
Gender				
Male	2.76 ± 0.44	2.3 ± 0.35	0.97 ± 0.18	1 ± 0.04
Female	2.8 ± 0.52	2.3 ± 0.39	1 ± 0.13	0.99 ± 0.05
Obesity				
Non-obese	2.6 ± 0.23 (c)	2.1 ± 0.39	1.02 ± 0.12	1.01 ± 0.01
Overweight	2.8 ± 0.37 (c)	2.28 ± 0.40	0.95 ± 0.21	0.991 ± 0.07
Obese	3.5 ± 0.58 (ab)	2.3 ± 0.17	1.01 ± 0.06	0.995 ± 0.04
Treatment with antidiabetic drugs				
Rosiglitazone	2.8 ± 0.45	2.3 ± 0.23	−	−
Glyburide	2.76 ± 0.5	2.2 ± 0.23		
Metformin	−	2.2 ± 0.23		

*DN* diabetic nephropathy, *DM* diabetes mellitus without nephropathy, *ND CKD* Nondiabetic chronic kidney disease, *a* significant differences compared to non-obese, *b* significant differences compared to overweight, *c* significant differences compared to obese

### The relation between *hsa-miR-221* and levels of albuminuria in the DN group

As shown in Fig. 1b, in diabetic patients with nephropathy, the mean levels of *hsa-miR-221* were significantly higher (P < 0.001) in patients with macroalbuminuria (3.11 ± 0.43) than those with microalbuminuria (2.43 ± 0.14). However, no considerable variation was found in the mean levels of *hsa-miR-221* between patients with macroalbuminuria (0.992 ± 0.03) and those with microalbuminuria (0.996 ± 0.2) in the nondiabetic CKD group (Fig. 1c).

### The significant correlations of serum *hsa-miR-221 *in the study

**Fig. 2:**
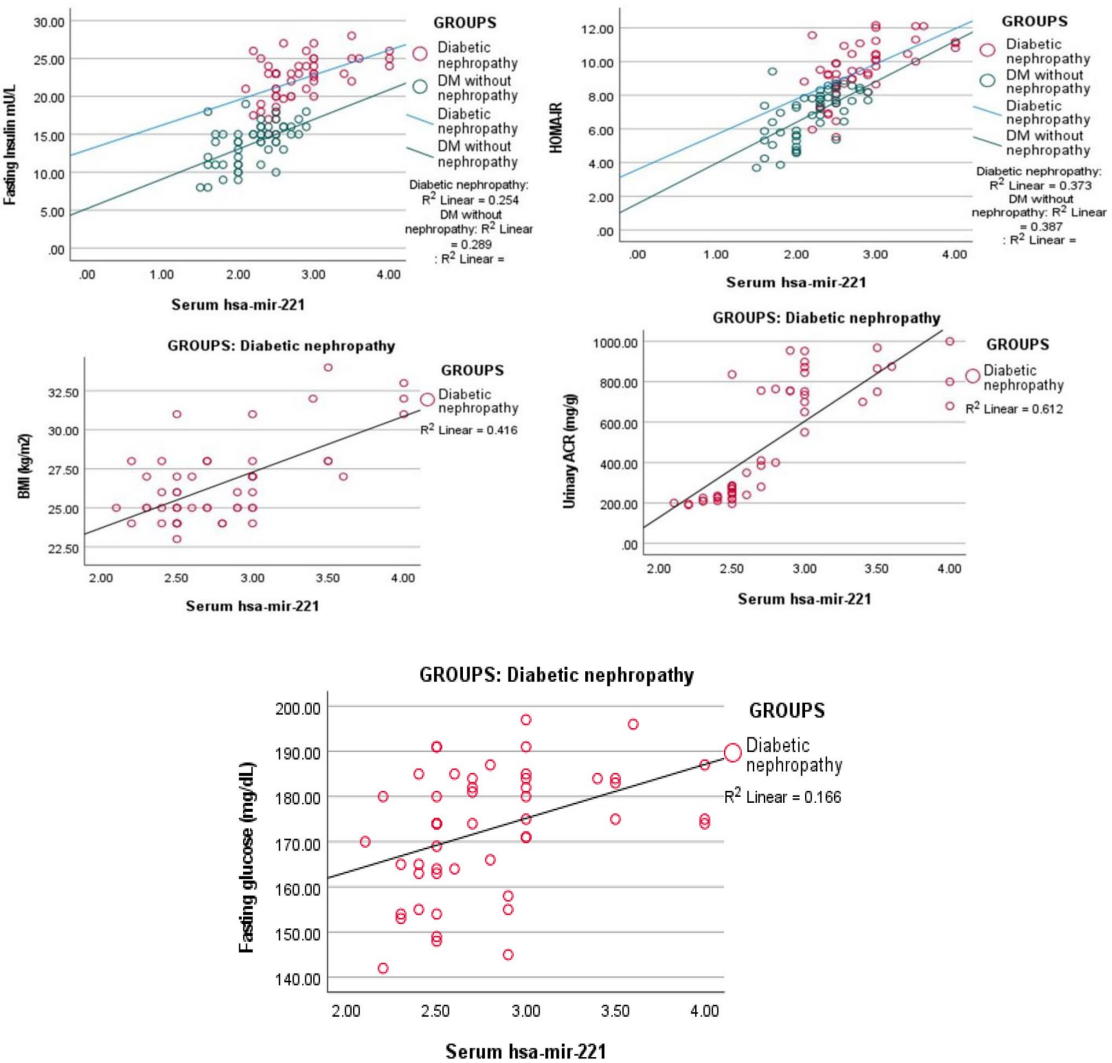
Significant correlations of serum *hsa-mir-221* in the studied groups. *Red dots* diabetic nephropathy group, *green dots* diabetic without nephropathy group

 According to Fig. 2, significant (P < 0.001) positive correlations were noticed between serum *hsa-miR-221* and fasting insulin (r = 0.504), fasting glucose (r = 0.407), HOMA IR (r = 0.610), ACR (r = 0.782), and BMI (r = 0.645) in diabetic nephropathy group. In diabetic patients without nephropathy, there were significant (P < 0.001) positive correlations between serum *hsa-miR-221* and fasting insulin (r = 0.537) and HOMA IR (r = 0.622) only.

### Logistic regression of significant predictor parameters for prediction of diabetic nephropathy

Univariate logistic regression revealed *hsa-miR-221* to be a significant predictor of the development of diabetic nephropathy in diabetic patients, along with HbA1c, fasting glucose, fasting insulin, HOMA IR, and BMI (Table 4). This result remained in multivariate logistic regression models, whether conventional risk factors (BMI) or the significant variables from the univariate models (*hsa-miR-221*, HbA1c, fasting glucose, and HOMA IR; Table 4) were specified as covariates. According to Table 4, the combination of *hsa-miR-221* and either fasting glucose or HbA1c showed the best performance and explained 70% and 79% (Nagelkerke R2) of the variation, respectively.

**Table 4:** Logistic regression of significant predictor parameters for prediction of diabetic nephropathy

	Significant predictor variables	Wald test	P value	OR	CI 95% EXP(B)	Nagelkerke R2 (%)
Univariate logistic regression	HbA1c	28.8	< 0.001	23.1	7–73	62
	Fasting glucose	21.7	< 0.001	0.92	0.89–0.95	48
	Fasting insulin	18.5	< 0.001	2.9	1.8–4.6	85
	HOMA IR	24	< 0.001	3.05	2–4.8	54
	BMI	13.6	< 0.001	1.6	1.2–2	21
	*hsa-miR-221*	21	< 0.001	57.8	10–327	47
Multivariate logistic regression models	*hsa-miR-221*	8	0.005	31	3.7–37	70
	HbA1c	17	< 0.001	12	2.9–332	
	*hsa-miR-221*	16.2	< 0.001	0.895	0.85–0.94	79
	Fasting glucose	16.5	< 0.001	329	19–5529	
	*hsa-miR-221*	4.4	0.04	8.6	1.1–64	58
	HOMA IR	10.3	0.001	2.3	1.4–3.8	
	*hsa-miR-221*	17.8	< 0.001	61	9–413	52
	BMI	5.4	0.021	1.4	1–1.9	

*Wald* Wald Chi-square, *OR* Odds Ratio (per increase of 1 SD of the parameter), *CI* Confidence interval

### ROC curve analysis of serum *hsa-miR-221 *in the early detection of diabetic nephropathy in diabetes mellitus

**Fig. 3:**
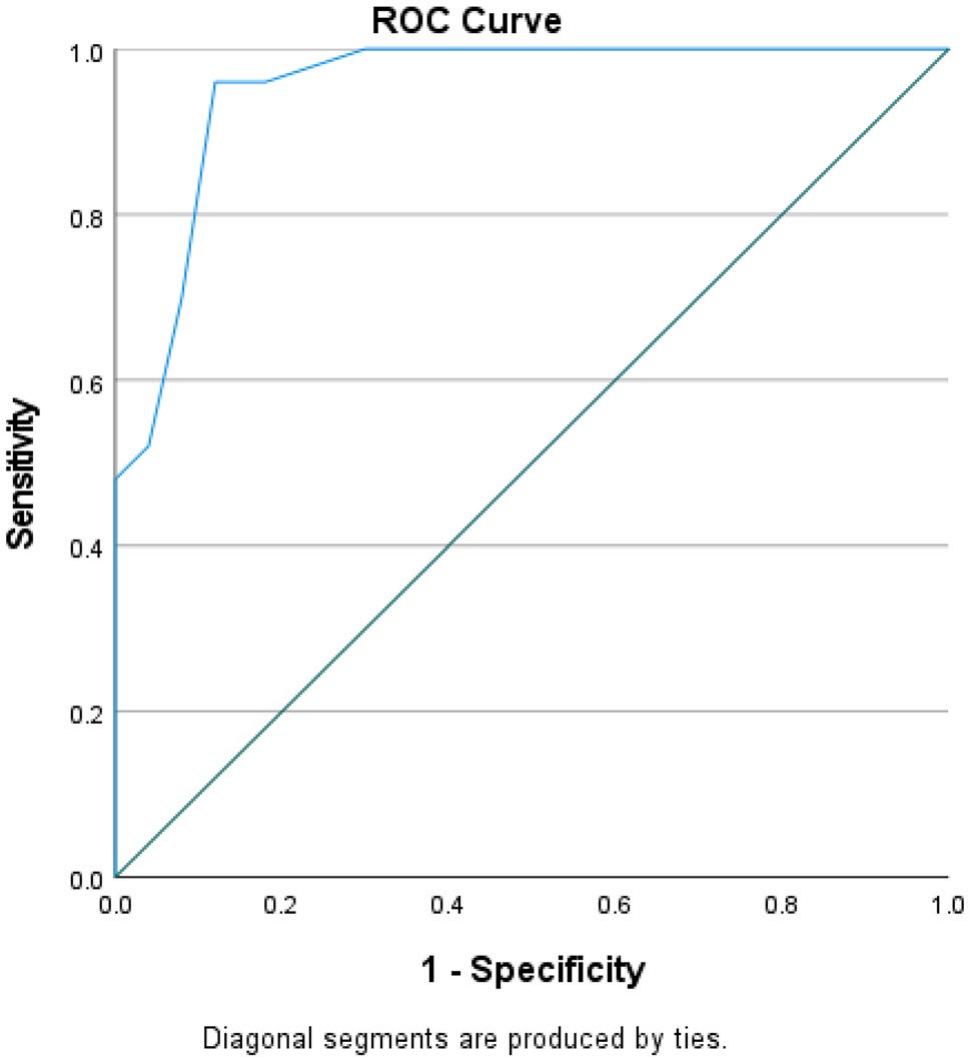
ROC Curve Analysis of serum *hsa-mir-221* in early detection of diabetic nephropathy in diabetic patients

The AUC of serum *hsa-miR-221* was 0.953 with a sensitivity of 96% and specificity of 88% at the cut-off value ≥ 2.55, with a significantly accurate diagnosis for patients with diabetic nephropathy, as shown in Fig. 3.

## Discussion

Early diagnosis and management of diabetic nephropathy are urgent to avoid complications and fatality [24].

Although albuminuria is considered the best available test for the diagnosis of diabetic nephropathy, additional measures, such as serum creatinine, urine ACR, or eGFR, can be used to assess renal damage [25]. However, none of those tests offers a significant sensitivity for early diagnosis of all patients with diabetic renal dysfunction [26, 27].

The current investigations demonstrated that the mean levels of BMI, serum fasting insulin, serum fasting glucose, HOMA IR, serum HbA1c, serum creatinine, and urinary ACR were significantly higher. In contrast, the mean eGFR concentrations were significantly decreased in diabetic nephropathy patients than their corresponding levels in diabetic patients with no nephropathy, nondiabetic patients with CKD, and healthy controls, indicating the increased severity of diabetes and the deterioration of the kidney in patients with diabetic nephropathy.

Although BUN levels were deemed significantly higher in DN than in diabetic patients without nephropathy, and healthy controls, they were comparable to nondiabetic patients with CKD, undistinguishing the kidney injury in both diseases.

In the present study, the *hsa-miR-221* mean levels revealed a significant increase in diabetic nephropathy and diabetic patients without nephropathy compared to patients with nondiabetic CKD and healthy controls. At the same time, they were higher in DN than in diabetic patients without nephropathy and comparable between nondiabetic CKD and healthy controls. These findings suggested the significance of *hsa-miR-221* for the diabetes pathogenesis and the progression of diabetic nephropathy.

This role was explained by Li et al. [28], and Poliseno et al. [11] who coincided with these results as they reported that longstanding hyperglycemia in diabetes resulted in induction of the expression of *hsa-miR-221* and dysfunction of endothelial cells. Also, Fiorentino et al. [29], Coinciding with the present findings Costantino et al. [30], Lightell et al. [13], and Li et al. [31] revealed that *hsa-miR-221* expression was highly increased in diabetes and might be included in the physiopathology of macrovascular and diabetes complications via specific genes.

Similar findings were found in diabetic retinopathy (DR), as researchers identified *hsa-miR-221* as a biomarker for DR in T2DM and proliferative DR patients, and it was thought to contribute to DM pathogenesis and associated macrovascular complications [32–35].

Also, Li et al., [28] explained the importance of *hsa-miR-221* in the occurrence and progression of T2DM as they reported that under high glucose levels, significantly elevated *hsa-miR-221* could promote apoptosis and cell proliferation facilitating endothelial dysfunction and hyperplasia of retinal vessels contributing to diabetic microvascular complications. As a result, *hsa-miR-221* seems to be abnormally increased in the serum of T2D patients, rising gradually as DR severity increased, aggravating the damage of retinal vessels and dysfunction exposed to elevated glucose levels suggesting that *hsa-miR-221* could be implicated in angiogenesis, proposing an innovative hypothesis for DR in T2D patients.

In contrast to earlier and current findings, Togliatto et al. [36] discovered downregulation of *hsa-miR-221* expression related to reduced angiogenesis in hyperglycemia, and Marei et al. [37] found substantial decreases in *hsa-miR-221* levels in both placenta and serum in the GDM group relative to the control group. The findings on the effect of glucose levels on *hsa-miR-221* expression are contentious. However, this could be attributed to modifications in cell culture procedures. Evidently, more research into the expression of *hsa-miR-221* in hyperglycemia is needed.

The current study found that the mean levels of *hsa-miR-221* in diabetic patients with nephropathy were greater in patients with macroalbuminuria than those with microalbuminuria but comparable in similar patients in the nondiabetic CKD group. Additionally, a significant positive correlation was noticed between *hsa-miR-221* and urinary ACR in diabetic patients with nephropathy.

The current outcomes agree with those of Atya et al. [38], who discovered that the serum *hsa-miR-221* expression and ACR ratio had a significant positive correlation in diabetic patients and patients with either proliferative or non-proliferative diabetic retinopathy.

The coincidence between *hsa-miR-221* and DN and the level of albuminuria was explained by Su et al. [39], who reported that the proximal tubule was the target of impact in DN. In high-glucose conditions, podocytes triggered the division of epithelial cells of the proximal tubule, and extracellular vehicles mediated the connection. The level of *hsa-miR-221*, which was extracted from these extracellular vehicles, was significantly higher, promoting the division of the proximal tubule’s epithelial cells. Additionally, in diabetic mice, the suppression of *hsa-miR-221* corrected these abnormal changes in the proximal tubule’s epithelial cells.

The present investigation found that in the diabetic patients with the nephropathy group, the *hsa-miR-221* concentrations were positively correlated with BMI, fasting insulin, fasting glucose, HOMA IR, and urinary ACR. In contrast, in the diabetic patients without the nephropathy group, these correlations were limited to fasting insulin and HOMA IR.

The current correlations of hsa-miR-221 were similarly reported by Lustig et al. [40] who discovered that *hsa-miR-221* was raised in obesity animal models, Atya et al. [38], who discovered that the serum *hsa-miR-221* expression was positively correlated with HBA1c and HOMA-IR in diabetic patients without retinopathy and those with different degrees of diabetic retinopathy compared to healthy subjects, Liu et al. [14] who discovered that the serum *hsa-miR-221* was significantly positively associated with metabolic HOMA IR in type 2 diabetes patients with diabetic retinopathy.

The explanation of these correlations were demonstrated by previous research [12], which revealed the binding of *hsa-miR-221* to important genes and components of the insulin/insulin-like signaling pathway, as well as their relationship with metabolic syndrome, diabetes, and diabetic implications, Lustig et al. [40], who revealed that *hsa-miR-221* could have a role in IR regulation via reducing adiponectin expression, and Meerson et al. [41] who showed that *hsa-miR-221* expression in subcutaneous adipose tissue was positively linked with BMI in nondiabetic Pima Indians.

In contrast to the present results, Chou et al. [42] found that *hsa-miR-221* concentrations in adipocytes were negatively associated with BMI. Additionally, Wang et al. [43] showed that metabolic syndrome-affected females without obesity had greater *hsa-miR-221* plasma concentrations than did controls, while Ortega et al. [44] found lower plasma concentrations of *hsa-miR-221* in obese patients.

The variance in these outcomes could be attributed to methodologic discrepancies or unknown attributes in the cohorts. Despite contradictory human data, *hsa-miR-221* can promote IR development by suppressing adiponectin signaling and has the potential as a circulating biomarker.

This study revealed considerable declines in the HDL-c serum levels along with considerable elevations in the TG and cholesterol serum levels in diabetic nephropathy and diabetic patients without nephropathy relative to nondiabetic patients with CKD and controls.

Similar findings were discovered by Vinod et al. [45], who reported that serum cholesterol and TG levels were significantly elevated while HDL levels were significantly decreased in patients with poor control of hyperglycemia than those with good control, implying that the lipid profile could be used to predict and monitor diabetes mellitus.

The ROC curve analysis for the role of serum *hsa-miR-221* in the early identification of diabetic nephropathy in diabetes mellitus revealed high substantial sensitivity and specificity with highly accurate diabetic nephropathy diagnosis.

No previous study investigated the use of serum *hsa-miR-221* in the early detection of diabetic nephropathy in diabetes mellitus. Still, Atya et al. [38], and Liu et al. [14] discovered related results in diabetic retinopathy, revealing that *hsa-miR-221* had clear diagnostic value in diabetic retinopathy in diabetic patients using ROC curve analysis.

By using the logistic regression, *hsa-miR-221* was found to be a significant predictor of the development of diabetic nephropathy in diabetic patients, along with HbA1c, fasting glucose, fasting insulin, HOMA IR, and BMI. The combination of *hsa-miR-221* and either fasting glucose or HbA1c showed the best performance and explained 70% and 79% of the variation, respectively assisting in an early diagnosis of the diabetic nephropathy, before kidney damage progresses or even before albuminuria develops.

## Conclusion

According to the current study, diabetic nephropathy patients had elevated levels of *hsa-miR-221* in their serum than diabetic patients without nephropathy and controls. In contrast, all diabetic patients had higher levels than nondiabetic individuals. Additionally, diabetic nephropathy patients with macroalbuminuria had increased levels of *hsa-miR-221* than those with microalbuminuria. The levels of *hsa-miR-221* positively correlated with the BMI, serum fasting insulin, serum fasting glucose, HOMA IR, and urinary ACR in the diabetic nephropathy group. The early detection of diabetic nephropathy in diabetes mellitus using serum *hsa-miR-221* exhibited significant sensitivity and specificity with an extremely accurate diagnosis of diabetic nephropathy. This study points to *hsa-miR-221* as a possible biomarker for diagnostic and prognostic proposes in diabetic nephropathy, contributing to diagnose the condition early before kidney damage worsens or even before albuminuria occurs. Future studies should pay greater focus to investigate novel methods to suppress the negative miRNAs. Prior to kidney structural pathological changes, therapeutic targeting of miRNAs would try to reverse these earlier changes.

## Data Availability

The corresponding author will provide the datasets produced and/or analysed during the current work upon reasonable request.

## Abbreviations

*Hsa*: *Homo sapiens*
*miR*: microRNA
CKD: Chronic kidney diseases
HOMA IR: Homeostatic Model Assessment for Insulin Resistance
ACR: Albumin to creatinine ratio
BMI: Body mass index

## References

[1] Sun H, Saeedi P, Karuranga S, Pinkepank M, Ogurtsova K, Duncan BB, Stein C, Basit A, Chan JC, Mbanya JC, Pavkov ME (2022). IDF diabetes atlas: global, regional and country-level diabetes prevalence estimates for 2021 and projections for 2045. Diabetes Res Clin Pract.

[2] Zhou B, Lu Y, Hajifathalian K, Bentham J, Di Cesare M, Danaei G, Bixby H, Cowan MJ, Ali MK, Taddei C, Lo WC (2016). Worldwide trends in diabetes since 1980: a pooled analysis of 751 population-based studies with 4.4 million participants. Lancet.

[3] Harding JL, Pavkov ME, Magliano DJ, Shaw JE, Gregg EW (2019). Global trends in diabetes complications: a review of current evidence. Diabetologia.

[4] Zhang XX, Kong J, Yun K (2020) Prevalence of diabetic nephropathy among patients with type 2 diabetes mellitus in China: a meta-analysis of observational studies. J Diabetes Res 2020 10.1155/2020/2315607 PMC7023800 32090116

[5] Samsu N (2021) Diabetic nephropathy: challenges in pathogenesis, diagnosis, and treatment. BioMed Res Int 2021 10.1155/2021/1497449 PMC8285185 34307650

[6] Yarahmadi A, Shahrokhi SZ, Mostafavi-Pour Z, Azarpira N (2021). MicroRNAs in diabetic nephropathy: from molecular mechanisms to new therapeutic targets of treatment. Biochem Pharmacol.

[7] United States Renal Data System (2022) 2022 USRDS annual data report: epidemiology of kidney disease in the United States. https://usrds-adr.niddk.nih.gov/2022/chronic-kidney-disease 10.1053/j.ajkd.2022.12.001 PMC10807034 36822739

[8] Dronavalli S, Duka I, Bakris GL (2008). The pathogenesis of diabetic nephropathy. Nat Clin Pract Endocrinol Metab.

[9] Wada J, Makino H (2013). Inflammation and the pathogenesis of diabetic nephropathy. Clin Sci.

[10] Kiyanpour F, Abedi M, Gheisari Y (2020) A systematic integrative approach reveals novel microRNAs in diabetic nephropathy. J Res Med Sci: Off J Isfahan Univ Med Sci 25 10.4103/jrms.JRMS_289_19 PMC7003547 32055241

[11] Poliseno L, Tuccoli A, Mariani L, Evangelista M, Citti L, Woods K, Mercatanti A, Hammond S, Rainaldi G (2006). MicroRNAs modulate the angiogenic properties of HUVECs. Blood.

[12] Deiuliis JA (2016). MicroRNAs as regulators of metabolic disease: pathophysiologic significance and emerging role as biomarkers and therapeutics. Int J Obes.

[13] Lightell DJ, Moss SC, Woods TC (2018). Upregulation of miR-221 and-222 in response to increased extracellular signal-regulated kinases 1/2 activity exacerbates neointimal hyperplasia in diabetes mellitus. Atherosclerosis.

[14] Liu HN, Li X, Wu N, Tong MM, Chen S, Zhu SS, Qian W, Chen XL (2018). Serum microRNA-221 as a biomarker for diabetic retinopathy in patients associated with type 2 diabetes. Int J Ophthalmol.

[15] Ashcroft RE (2008). The declaration of Helsinki. The Oxford textbook of clinical research ethics.

[16] Chamberlain JJ, Rhinehart AS, Shaefer CF, Neuman A (2016). Diagnosis and management of diabetes: synopsis of the 2016 American diabetes association standards of medical care in diabetes. Ann Intern Med.

[17] American Diabetes Association (2016). Microvascular complications and foot care. Diabetes Care.

[18] Keys A, Fidanza F, Karvonen MJ, Kimura N, Taylor HL (1972). Indices of relative weight and obesity. J Chronic Dis.

[19] Bonora E, Targher G, Alberiche M, Bonadonna RC, Saggiani F, Zenere MB, Monauni TI, Muggeo M (2000). Homeostasis model assessment closely mirrors the glucose clamp technique in the assessment of insulin sensitivity: studies in subjects with various degrees of glucose tolerance and insulin sensitivity. Diabetes Care.

[20] Romero-Gómez M, Viloria MD, Andrade RJ, Salmerón J, Diago M, Fernández-Rodríguez CM, Corpas R, Cruz M, Grande L, Vázquez L, Muñoz-de-Rueda P (2005). Insulin resistance impairs sustained response rate to peginterferon plus ribavirin in chronic hepatitis C patients. Gastroenterology.

[21] Levey AS, Stevens LA, Schmid CH, Zhang Y, Castro AF, Feldman HI, Kusek JW, Eggers P, Van Lente F, Greene T, Coresh J (2009). A new equation to estimate glomerular filtration rate. Ann Intern Med.

[22] Friedewald WT, Levy RI, Fredrickson DS (1972). Estimation of the concentration of low-density lipoprotein cholesterol in plasma, without use of the preparative ultracentrifuge. Clin Chem.

[23] Livak KJ, Schmittgen TD (2001). Analysis of relative gene expression data using real-time quantitative PCR and the 2 –. ∆∆CT method.

[24] American Diabetes Association (2020). 2. Classification and diagnosis of diabetes: standards of medical care in diabetes—2020. Diabetes Care.

[25] Liu R, Li G, Cui XF, Zhang DL, Yang QH, Mu XY, Pan WJ (2011). Methodological evaluation and comparison of five urinary albumin measurements. J Clin Lab Anal.

[26] Zhou B, Zou H, Xu G (2016). Clinical utility of serum cystatin c in predicting diabetic nephropathy among patients with diabetes mellitus: a meta-analysis. Kidney Blood Press Res.

[27] Levey AS, Coresh J, Tighiouart H, Greene T, Inker LA (2019). Strengths and limitations of estimated and measured GFR. Nat Rev Nephrol.

[28] Li Y, Song YH, Li F, Yang T, Lu YW, Geng YJ (2009). MicroRNA-221 regulates high glucose-induced endothelial dysfunction. Biochem Biophys Res Commun.

[29] Fiorentino L, Cavalera M, Mavilio M, Conserva F, Menghini R, Gesualdo L, Federici M (2013). Regulation of TIMP3 in diabetic nephropathy: a role for microRNAs. Acta Diabetol.

[30] Costantino S, Paneni F, Lüscher TF, Cosentino F (2016). MicroRNA profiling unveils hyperglycaemic memory in the diabetic heart. Eur Heart J.

[31] Li MY, Pan SR, Qiu A (2016). Roles of microRNA-221/222 in type 2 diabetic patients with post-menopausal breast cancer. Genet Mol Res.

[32] Martinez B, Peplow PV (2019). MicroRNAs as biomarkers of diabetic retinopathy and disease progression. Neural Regen Res.

[33] Mammadzada P, Bayle J, Gudmundsson J, Kvanta A, André H (2019). Identification of diagnostic and prognostic microRNAs for recurrent vitreous hemorrhage in patients with proliferative diabetic retinopathy. J Clin Med.

[34] Chen S, Yuan M, Liu Y, Zhao X, Lian P, Chen Y, Liu B, Lu L (2019). Landscape of microRNA in the aqueous humour of proliferative diabetic retinopathy as assessed by next-generation sequencing. Clin Exp Ophthalmol.

[35] Smit-McBride Z, Nguyen KN, Lai AW, Elliott GW, Nguyen JD, Nguyen AT, Morse LS (2018). The effect of DR circulatory microRNAs on VEGF secretion in human retinal pigment epithelial cells. Investig Ophthalmol Vis Sci.

[36] Togliatto G, Trombetta A, Dentelli P, Rosso A, Brizzi MF (2011). RETRACTED ARTICLE: MIR221/MIR222-driven post-transcriptional regulation of P27KIP1 and P57KIP2 is crucial for high-glucose-and AGE-mediated vascular cell damage. Diabetologia.

[37] Marei E, Gabr Youssef H (2020). Evaluation of MicroRNA-16 and MicroRNA-221 in serum and placenta in gestational diabetes mellitus: correlation with macrosomia. Egypt J Radiat Sci Appl.

[38] Atya SG, Elmohamady SN, Zidan MA, Fallah AA (2021). MicroRNA-221 in serum as a biomarker for diabetic retinopathy in type 2 diabetes Egyptian patients. Ann Rom Soc Cell Biol.

[39] Su H, Qiao J, Hu J, Li Y, Lin J, Yu Q, Zhen J, Ma Q, Wang Q, Lv Z, Wang R (2020). Podocyte-derived extracellular vesicles mediate renal proximal tubule cells dedifferentiation via microRNA-221 in diabetic nephropathy. Mol Cell Endocrinol.

[40] Lustig Y, Barhod E, Ashwal-Fluss R, Gordin R, Shomron N, Baruch-Umansky K, Hemi R, Karasik A, Kanety H (2014). RNA-binding protein PTB and microRNA-221 coregulate AdipoR1 translation and adiponectin signaling. Diabetes.

[41] Meerson A, Traurig M, Ossowski V, Fleming JM, Mullins M, Baier LJ (2013). Human adipose microRNA-221 is upregulated in obesity and affects fat metabolism downstream of leptin and TNF-α. Diabetologia.

[42] Chou WW, Wang YT, Liao YC, Chuang SC, Wang SN, Juo SH (2013). Decreased microRNA-221 is associated with high levels of TNF-α in human adipose tissue-derived mesenchymal stem cells from obese woman. Cell Physiol Biochem.

[43] Wang YT, Tsai PC, Liao YC, Hsu CY, Juo SH (2013). Circulating microRNAs have a sex-specific association with metabolic syndrome. J Biomed Sci.

[44] Ortega FJ, Mercader JM, Catalan V, Moreno-Navarrete JM, Pueyo N, Sabater M, Gomez-Ambrosi J, Anglada R, Fernández-Formoso JA, Ricart W, Frühbeck G (2013). Targeting the circulating microRNA signature of obesity. Clin Chem.

[45] VinodMahato R, Gyawali P, Raut PP, Regmi P, Singh KP, Pandeya DR, Gyawali P (2011). Association between glycaemic control and serum lipid profile in type 2 diabetic patients: glycated haemoglobin as a dual biomarker. Biomed Res.

