# Correction: Circulating *hsa-miR-221* as a possible diagnostic and prognostic biomarker of diabetic nephropathy

**DOI:** 10.1007/s11033-023-09085-x

**Published:** 2023-12-20

**Authors:** Marwa Sayed Abdel-Tawab, Mohamed Gamal Mohamed, Noha A. Doudar, Enas Ezzat Rateb, Hoda Ramadan Reyad, Naglaa Adli Abd Elazeem

**Affiliations:** 1 Medical Biochemistry Department, Faculty of Medicine, Beni-Suef University, Beni-Suef, Egypt; 2 Internal Medicine Department, Faculty of Medicine, Beni-Suef University, Beni-Suef, Egypt; 3 Clinical and Chemical Pathology Department, Faculty of Medicine, Beni-Suef University, Beni-Suef, Egypt; 4 Physiology Department, Faculty of Medicine, Beni-Suef University, Beni-Suef, Egypt

**Correction to: Molecular Biology Reports (2023) 50:9793–9803** 10.1007/s11033-023-08846-y

In Table 1 of this article, the data in the row with the heading “Macro-albuminuria” and columns “DM N = 50 N/%” and “Controls N = 50 N/%” were incorrectly listed as “ − / − 50/100%”. It should have been “-/- “.

In the row with the heading “Normal”, the data in the columns “DM N = 50 N/%” and “Controls N = 50 N/%” are empty. It should have been “50/100%”. The correct Table 1 is given below.

**Table 1:** The frequency of some demographic, and clinical data in the studied groups

Parameters	DN N = 50 N/%	DM N = 50 N/%	ND CKD N = 50 N/%	Controls N = 50 N/%
Gender				
Male	28/56%	29/58%	31/62%	33/66%
Female	22/44%	21/42%	19/38%	17/34%
Obesity				
Non-Obese	10/20%	27/54%	26/52%	42/84%
Overweight	33/66%	17/34%	20/40%	6/12%
Obese	7/14%	6/12%	4/8%	2/4%
Albuminuria				
Micro-albuminuria	23/46%	−/−	30/60%	−/−
Macro-albuminuria	27/54%	−/−	20/40%	−/−
Normal	−/−	50/100%	−/−	50/100%
Treatment with antidiabetic drugs				
Rosiglitazone	24/48%	22/44%	−/−	−/−
Glyburide	26/52%	14/28%	−/−	−/−
Metformin	−/−	14/28%	−/−	−/−

*DN* diabetic nephropathy, *DM* diabetes mellitus without nephropathy, *ND CKD* Nondiabetic chronic kidney disease, *N* number of cases, *%* percentage of frequency

The original article has been corrected.

